# Lactate of Beta-Eucain

**Published:** 1905-05

**Authors:** 


### Lactate of Beta-eucain.

H. Bert Ellis, Professor of Ophthalmology, University of Southern California, Los Angeles, read a report on "The Use of Beta-eucain Lactate in Eye, Nose and Throat Work," before the Los Angeles County Medical Association, February 3, 1905, (*California Med. and Surg. Reporter,* March 5, 1905,) in which he said: I have been using 12 per cent. solutions of the lactate of beta-eucain to anesthetise the mucous membrane in making diagnosis of nose, throat and ear troubles. It does not disturb the circulation nor shrink the tissues, so that the tissues may be seen with the probe while in their natural condition. For removing portions of the turbinated bodies I use a tampon of cotton saturated with 12 per cent. solution, allow it to remain five minutes, replace it with another and allow it also to remain five minutes. Then, ordinarily, the parts are ready for operation.

When spurs of the septum are to be removed, after using the lactate as above, I put in a tampon saturated with epinephrin 1-10000, so the tissues will be shrunk as much as possible. Epinephrin may also be used in combination with the lactate when operating on the middle turbinated bodies or the ethmoidal cells.

A 5 per cent. solution of the lactate of beta-eucain, when dropped into the conjunctival sac, causes a more decided smarting than does a 4 per cent. solution of cocain, but it creates neither hyperemia nor anemia and it has the decided advantage over cocain that it in no way disturbs vision, having neither cycloplegic nor mydriatic action. The anesthetic effect is excellent and quite equal to that of a 4 or 5 per cent. cocain solution, and it produces no lesion of the cornea.

By infiltration the following will in the course of thirty minutes effect an anesthesia which lasts three or four hours. There are no depressing effects, even if twice the quantity is injected.

| | | |
|---|---|---|
| ℞ | Lactate of beta-eucain . . . . . . | gr. 4 |
| | Sodium chloride, c. p. . . . . . . | gr. 12 |
| | Sol. epinephrin (1-1000) . . . . . | m. 10 |
| | Distilled water . . . . . . . | oz. 3½ |

Inject one-half for ordinary operations.

